# The female mouse is resistant to mild vitamin B_3_ deficiency

**DOI:** 10.1007/s00394-021-02651-8

**Published:** 2021-08-02

**Authors:** Inge van der Stelt, Wenbiao Shi, Melissa Bekkenkamp-Grovenstein, Rubén Zapata-Pérez, Riekelt H. Houtkooper, Vincent C. J. de Boer, Maria A. Hegeman, Jaap Keijer

**Affiliations:** 1 Human and Animal Physiology, Wageningen University, PO Box 338, 6700 AH Wageningen, The Netherlands; 2 Present Address: Key Laboratory of Precision Nutrition and Food Quality, Department of Nutrition and Health, China Agricultural University, Beijing, 100083 China; 3 Laboratory Genetic Metabolic Diseases, Amsterdam Gastroenterology, Endocrinology, and Metabolism, Amsterdam Cardiovascular Sciences, Amsterdam UMC, University of Amsterdam, Meibergdreef 9, 1105 AZ Amsterdam, The Netherlands; 4 Present Address: Educational Consultancy and Professional Development, Faculty of Social and Behavioural Sciences, Utrecht University, 3584 CS Utrecht, The Netherlands

**Keywords:** Vitamin B_3_ deficiency, Nicotinamide riboside, Tryptophan, Male–female differences, Insulin sensitivity, White adipose tissue

## Abstract

**Purpose:**

Vitamin B_3_ provides nicotinamide adenine dinucleotide (NAD^+^), an essential coenzyme in oxidoreductase reactions. Severe vitamin B_3_ deficiency leads to the disease Pellagra, while mild vitamin B_3_ deficiency has been linked to age-related and metabolic diseases. Mild vitamin B_3_ deficiency is understudied, especially in females. Therefore, we examined how female mice responded to a diet that induced mild vitamin B_3_ deficiency in male mice.

**Methods:**

Female C57BL/6RccHsd mice were subjected for 18 weeks to a diet without vitamin B_3_ and low but sufficient tryptophan (0.115%) (0NR) and were compared to control female mice on the same diet with the reference dose of vitamin B_3_ (30NR, 30 mg nicotinamide riboside/ kg diet).

**Results:**

In the female mice, no differences between the two dietary groups were found in liver nicotinamide mononucleotide (NMN) levels, body composition, whole body energy and substrate metabolism measured by indirect calorimetry, or liver triacylglycerol metabolism. Expression of seven genes that previously were shown to respond to mild vitamin B_3_ deficiency in male white adipose tissue were not differentially expressed between the female dietary groups, neither was insulin sensitivity.

**Conclusion:**

We concluded that the female 0NR mice were not vitamin B_3_ deficient; the role of age, sex and health status is discussed. Demonstrated by clear differences between females and males, the latter showing mild deficiency under the same conditions, this study highlights the importance of studying both sexes.

**Supplementary Information:**

The online version contains supplementary material available at 10.1007/s00394-021-02651-8.

## Introduction

Oxidised nicotinamide adenine dinucleotide (NAD^+^) is essential as a coenzyme in oxidoreductase reactions, and it functions as a consumed ligand in, for example, sirtuin- and poly(ADP-ribose) polymerase-related activities [1, 2]. Dietary intake of vitamin B_3_, which exists in various forms, and de novo synthesis from the essential amino acid tryptophan, via the kynurenine pathway, are of essence to produce NAD^+^. Nicotinic acid and nicotinamide are forms of vitamin B_3_ which can generate NAD^+^ via the Preiss-handler pathway and the NAD^+^ salvage pathway, respectively; the more recently discovered nicotinamide riboside (NR) shares metabolic steps with nicotinamide, however, distinctly using nicotinamide riboside kinase 1 or 2 (NRK1/2) [3, 4].

Vitamin B_3_ has gained interest since it was shown that an increased intake improved health, for example, in hypercholesterolaemic patients. Nicotinic acid supplementation was reported in human studies to reduce, amongst other parameters, circulating levels of total cholesterol, LDL to HDL ratio, and triacylglycerols [5–9] towards a more healthy lipid profile. Using a partly different pathway compared to nicotinic acid expected to result in less side effects, NR supplementation showed a potent increase in (mitochondrial) NAD^+^ levels in vitro and in vivo [10]. Amongst other parameters, the liver of mice fed a high-fat diet supplemented with a high dose of NR (400 mg/kg/day) showed a 40% reduction in triacylglycerol content, and decreased cholesterol levels were found, resulting in an improved lipid profile in the NR supplemented mice [10].

Contrasting vitamin B_3_ supplementation, severe vitamin B_3_ deficiency results in Pellagra, a disease characterised by dermatitis, diarrhoea, dementia, and finally death [11, 12]. Although mostly prevented by education on food choices and food preparation, the disease still exists in poorer countries and in patients with severe malnutrition due to, for example, anorexia or severe alcoholism. Effects of mild, subclinical deficiency are less clear, but may be relevant on a population level, as it may contribute to metabolic diseases as well as age-related diseases. Mild vitamin B_3_ deficiency, not inducing Pellagra, was, for example, shown to affect insulin sensitivity [13]. Next to this, the discovered age-related decline in NAD^+^ levels has been related to age-related diseases such as Alzheimer’s disease, where treatment with vitamin B_3_ showed beneficial effects [14–16]. Therefore, it is important to understand, and hence investigate, the effects of a mild vitamin B_3_ deficiency, and to discover its early signs. In a recent study in male C57BL/6JRccHsd mice, we found that mild vitamin B_3_ deficiency emerged after 18 weeks on a diet without vitamin B_3_. In particular, in the male mice on the deficient diet insulin sensitivity was decreased, liver nicotinamide mononucleotide (NMN) levels were lower and in white adipose tissue seven genes were differentially expressed, compared to male mice on the same diet with vitamin B_3_ [17]. In this study, NR was used as vitamin B_3_ in the control diet at the AIN93 recommended level (30 mg/kg diet) [18], which we recently confirmed as optimal dose to support health in mice [13].

While vitamin B_3_ deficiency studies are rare, this is even more true for female mice, despite marked metabolic differences between the sexes [19, 20]. Therefore, with this study, we wanted to increase the rather poor knowledge of a mild vitamin B_3_ deficiency and specifically gain knowledge on females. We subjected female mice to the exact circumstances causing mild vitamin B_3_ deficiency in male mice. We measured body composition and related parameters, energy metabolism and metabolic flexibility. Next to this, we analysed if the positive relation between lipid profile and vitamin B_3_ supplementation, as found by others, holds in case of a mild vitamin B_3_ deficiency; i.e. whether a worsening of the lipid profile occurred. We investigated the expression of seven genes in the white adipose tissue of the female mice that were previously found to be responsive to mild vitamin B_3_ deficiency in males. To define the NAD^+^ status, we measured the abundance of NAD^+^ and related metabolites in liver. Lastly, insulin sensitivity was determined in the females and compared to the male insulin sensitivity parameters.

## Materials and methods

### Animal study

The animal experiment was ethically approved (DEC2016033b) and performed in full accordance with national and EU regulations. This independent experiment was performed in parallel with an experiment with male mice [17], in which the exact same conditions induced a mild vitamin B_3_ deficiency. Twenty-four C57BL/6JRccHsd female mice (Envigo, Horst, the Netherlands) were individually housed (12 h light–dark cycle, 23 ± 1 °C, 55 ± 15% humidity), with ad libitum access to feed and water, unless indicated otherwise. Eleven-week-old mice were accustomed for 2 weeks to the control (30NR) moderately high-fat diet containing 40% energy from fat, 41% energy from carbohydrates, and 19% energy from proteins, and importantly 30 mg NR per kg diet as vitamin B_3_ source combined with a low but sufficient level (0.115%) of l-tryptophan (Research Diet Services, Wijk bij Duurstede, the Netherlands, see supplementary Table 1 for full ingredient information). Subsequently, mice were stratified based on body weight into two experimental groups (*n* = 12) which received either the control 30NR diet (30NR) or the same diet, except that NR was omitted (0NR). Intended to study recovery from vitamin B_3_ deficiency, an additional group fed the 0NR diet for 15 weeks followed by the 30NR diet during the last 3 weeks (0 + 30NR) was also studied (*n* = 12). Body weight and feed intake as well as lean and fat mass (by NMR, EchoMRI, Houston, USA) were measured weekly. Indirect calorimetry was performed in week 14, and an oral glucose tolerance test was conducted in week 17; blood and plasma were collected for glucose and insulin measurements, respectively. Mice were killed after 18 weeks on the diet by decapitation in a fed state, i.e. four hours after refeeding with 1.8 g of diet at the start of the light phase. Whole blood was collected in serum tubes (Greiner Bio-one, Longwood, USA), and serum was obtained by centrifugation at 3000*g*, 4 °C for 10 min, aliquoted and stored at − 80 °C. White adipose tissue and liver were snap frozen into liquid nitrogen and stored at − 80 °C.

### Indirect calorimetry

Oxygen (O_2_) consumption and carbon dioxide (CO_2_), hydrogen and methane production were measured in week 14 using a PhenoMaster System (TSE Systems, Bad Homburg, Germany), as described [13, 21]. Respiratory exchange ratio (RER) and energy expenditure were obtained using TSE software. Respiratory exchange ratio (RER) was defined as CO_2_ production (VCO2) divided by O_2_ consumption (VO_2_), and energy expenditure (kcal/h) was calculated with TSE Software 4.2.3 using the equation: Energy expenditure = [3.941 × VO_2_ + 1.106 × VCO_2_]/1000. After an adaptation period of 20 h, the mice were measured during ad libitum feeding for 24 h. Then, a fasting and refeeding challenge was conducted to assess metabolic flexibility based on the change in RER. The mice were given 1.5 g of the experimental diet (0NR or 30NR, depending on the experimental group) right before the dark period, which led to a fasted state during the light phase, where the response to refeeding was examined by providing the mice with 1.8 g of the corresponding experimental diet. ΔRER was calculated by subtracting average fasted RER (7:00–16:00) from average refed RER (17:30 h–01:00).

### Oral glucose tolerance test

An oral glucose tolerance test was performed in week 17, as described [21]. Briefly, after 6 h fasting, blood glucose was measured using blood from the distal end of the tail of the mice, at time point *t* = 0 and after oral glucose gavage (2 g/kg body weight) at *t* = 15, 30, 60, 90, and 120 min using a Freestyle blood glucose metre (Abbott Diabetes Care, Hoofddorp, The Netherlands). Plasma was collected at *t* = 0, 15, and 30 min using Microvette CB 300 tubes with potassium EDTA (1.6 mg EDTA/mL blood, Sarstedt, Etten-Leur, The Netherlands), which was centrifugated for 20 min at 2000*g*, 4 °C. This plasma was the input for the mouse insulin ELISA kit (Crystal Chem, Downers Grove, USA), measuring insulin according to instructions. To assess insulin sensitivity, homeostatic model assessment of insulin resistance (HOMA-IR) was used and calculated as (fasting glucose in mmol/L × fasting insulin in mU/L)/14.1 [22].

### Triacylglycerols and free fatty acids

Serum free fatty acids were measured in duplicate using a NEFA-HR kit (Wako chemical GmbH, Neuss, Germany) according to the manufacturer’s instructions. In short, 5 µl serum was incubated with 200 µl reagent R1 for 10 min at 37 °C, which was subsequently incubated with 100 µl reagent R2 for 10 min at 37 °C, after which absorbance at 546 nm was measured using a synergy HT microplate reader (BioTek instruments Inc., Winooski, VT, USA) and corrected for background absorbance at 660 nm. Standard curves of NEFA standard solution (Wako chemical GmbH) were used for quantification of free fatty acids. Liver triacylglycerols were measured in triplicate using a triglyceride liquicolor mono kit (Human, Wiesbaden, Germany), according to the manufacturer’s instructions. In short, liver was ground in liquid nitrogen and homogenised in homogenization buffer (10 mM Tris, 2 mM EDTA, and 250 mM sucrose). 4 µl of homogenate was incubated with 100 µl of reagent for 45 min, after which absorbance at 500 nm was measured using a synergy HT microplate reader (BioTek instruments Inc., Winooski, VT, USA). Standard curves of triacylglycerol standard supplied in the kit was used for quantification of triacylglycerol levels. Triacylglycerol levels were expressed per amount of total protein in the homogenates, which was measured using the DC-protein kit (Bio-rad).

### RNA Isolation and cDNA synthesis

Total RNA was isolated from white adipose tissue and liver with a RNeasy Mini kit (Qiagen, Venlo, the Netherlands), according to the manufacturer’s instructions. RNA yield and purity were checked using a Nanodrop spectrophometer (IsoGen Life Science, Maarsen, The Netherlands) and RNA integrity was verified using a TapeStation (Agilent, Santa Clara, CA, USA). One microgram of RNA was converted to cDNA using the iScript cDNA synthesis kit (Bio-Rad, Veenendaal, The Netherlands).

### Real time quantitative reverse transcription polymerase chain reaction (RT-qPCR)

RT-qPCR was performed using SYBR Green Supermix (Bio-Rad), according to the manufacturer’s instructions with an end volume of 25 μl, using a CFX96 real time PCR detection system (Bio-rad). Standard curves of pooled samples, negative controls, duplicates, melting profiles, R2 and PCR efficiency were used for validation of each run according to the MIQE guidelines [23].

Gene expression of the following genes was analysed in liver: apolipoprotein B (*Apob*), diacylglycerol O-acyltransferase 2 (*Dgat2*), microsomal triglycerol transfer protein (*Mttp*) using reference genes ribosomal protein S15 (*Rps15*), hypoxanthine guanine phosphoribosyl transferase (*Hprt*) and calnexin (*Canx*) for normalisation. In white adipose tissue, mRNA expression of the following genes was measured: acidic (leucine-rich) nuclear phosphoprotein 32 family, member A (*Anp32A*), mitogen-activated protein kinase kinase 1 (*Map2k1*), mitogen-activated protein kinase 1 (*Mapk1*), 5, 10-methenyltetrahydrofolate synthetase (*Mthfs*), 5, 10-methenyltetrahydrofolate synthetase-like (*Mthfsl*), quinoid dihydropteridine reductase (*Qdpr*), solute carrier family 2 (facilitated glucose transporter), member 4 (*Slc2a4*), tyrosine kinase, non-receptor, 2 (*Tnk2*) using reference genes beta-2 microglobin (*B2m*) and *Rps15*. An overview of all genes, primers and their sequences used can be found in Supplementary Table 2. Relative gene expression was expressed as the normalized expression values of the 0NR mice compared to the mean of the values of the 30NR control mice set at 1.

### Mass spectrometry measurements

Metabolomics analysis was performed as previously described with minor modifications [24]. 6–8 mg of freeze-dried liver was metabolically quenched using ice-cold methanol (500 µl) and diluted with Milli-Q water (500 µl) containing the internal standards, D_5_-glutamine, D_5_-phenylalanine, adenosine–^15^N_5_–monophosphate, adenosine–^15^N_5_–triphosphate, and guanine–^15^N_5_–triphosphate (5 μm each). Samples were then homogenised using a TissueLyser II (Qiagen, Hilden, Germany) for 5 min at a frequency of 30 times per second. Phase separation was obtained by chloroform addition (1 mL) followed by thorough mixing and centrifugation at 16,000*g* for 5 min at 4 °C. The polar upper phase was then transferred to 1.5 mL tubes and dried in a vacuum concentrator at 60 °C. The pellets obtained after concentration were dissolved in 100 µl methanol/water (6/4; *v*/*v*).

Metabolite analysis was performed using a Waters Acquity ultra-high performance liquid chromatography system coupled to a Bruker Impact II™ Ultra-High Resolution Qq-Time-Of-Flight mass spectrometer. Chromatography was performed in a SeQuant ZIC-cHILIC column (PEEK 100 × 2.1 mm, 3 µm particle size; Merck, Kenilworth, NJ, USA) at 30 °C. The LC method consisted in a gradient running at 0.25 mL/min from 100% mobile phase B (9:1 acetonitrile:water with 5 mM ammonium acetate pH 8.2) to 100% mobile phase A (1:9 acetonitrile:water with 5 mM ammonium acetate pH 6.8) in 28 min, followed by a re-equilibration step at 100% B of 5 min. Mass spectrometry data were acquired both in negative and positive ionisation modes over the range of *m*/*z* 50–1200. Data from full-scan mass spectrometry mode was analysed using Bruker TASQ software (Version 2.1.22.1 1065). All reported metabolite intensities were normalised to tissue weight, as well as to internal standards with comparable retention times and response in the mass spectrometer.

### Statistics

Graphpad Prism version 5.04 (Graphpad Software, San Diego, CA, USA) was used for statistical analysis. Comparisons between normally distributed 30NR and 0NR data were done with an unpaired Student’s *t*-test (body parameters, gene expression, blood parameters), when not normally distributed a Mann–Whitney *U* test was used. For measurements repeated over time two-way ANOVA was used with Bonferroni post-hoc testing (indirect calorimetry measurements, and oral glucose tolerance test). Differences were considered significant for *p* < 0.05.

## Results

### Body composition and food intake

Total body weight and body composition measured by echoMRI did not show statistical differences between the female mice supplied the diet with a reference amount of vitamin B_3_ (30NR; 30 mg NR/kg) and mice on the same diet but without vitamin B_3_ (0NR), both diets with a minimal (essential) level of tryptophan (Table 1). At the start of the experiment, body weight (21.0 g for 0NR and 20.7 g for 30NR, *p* = 0.544) and body composition (11.1% adiposity for 30NR and 11.2% adiposity for 0NR, *p* = 0.885) were not different nor were any differences observed at any of the intermediate timepoints (data not shown). No differences are seen in serum leptin levels at the end of the study (0.781 µg/mL for 30NR and 0.544 µg/mL for 0NR, *p* = 0.126), confirming that the diets did not affect fat mass. Similarly, total food intake was comparable between the 0NR and 30NR female mice. Therefore, it can be concluded that the depletion of NR from the diet for 18 weeks did not affect body composition and food intake in the female mice. Similarly, no obvious visual differences between the two dietary groups in behaviour or health were observed during the study and tissues appeared visually healthy upon section.

**Table 1:** Body composition and food intake of 30NR and 0NR mice at the end of the study

	30NR ± SD	0NR ± SD	*p* value
Body weight (g)	31.6 ± 4.6	29.4 ± 4.4	0.231
Lean mass (g)	19.1 ± 1.2	19.1 ± 1.2	0.946
Fat mass (g)	10.8 ± 3.8	8.6 ± 3.4	0.149
Adiposity (%)	33.3 ± 7.6	28.3 ± 7.6	0.127
Total food intake (g)	343.9 ± 31.4	327.3 ± 39.1	0.262

Endpoint measurements (*t* = 18 weeks) with no differences between body weight, fat mass, and lean mass. Adiposity is calculated using fat mass and body weight. Total food intake is the accumulated food intake over 18 weeks. 30NR mice (*n* = 12) had 30 mg/kg nicotinamide riboside in their diets, 0NR mice (*n* = 12) were on diets without nicotinamide riboside and other forms of vitamin B_3_. *SD* standard deviation

### Whole body metabolism

**Fig. 1:**
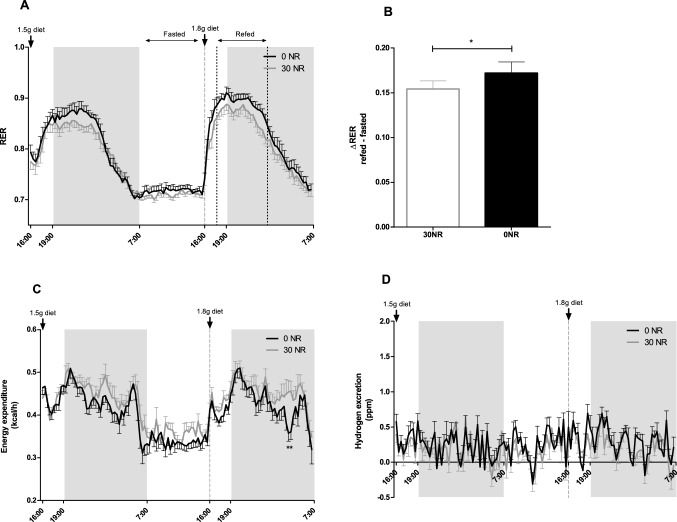
Indirect calorimetry measurements shows similar RER, energy expenditure and hydrogen excretion in mice on a 30 mg NR/kg diet (30NR) or no NR (0NR). **a** Respiratory exchange ratio (RER), **b** ΔRER, calculated by subtracting average refed RER (17:30 h–01:00) by average fasted RER (7:00–16:00), **c** Energy expenditure, **d** Hydrogen excretion, measured at week 14 of the study. Significant differences of *p* < 0.05 are indicated with *. Values are means ± SEM (*n* = 12)

The effect of omitting dietary NR for 18 weeks on whole body energy metabolism was measured using indirect calorimetry. Fat and carbohydrate oxidation, as measured by RER, did not show significant differences between 0 and 30NR female mice when analysed by two-way ANOVA (Fig. 1A), although metabolism of the 0NR mice tended to be skewed towards carbohydrate metabolism. The refeeding challenge, used as a measure for metabolic flexibility, was borderline significant (Fig. 1B). The energy expenditure was slightly lower in the 0NR mice, significant at two timepoints, however, ANOVA testing resulted in no overall difference between the two female dietary groups (Fig. 1C). Hydrogen excretion measurements were overall low, indicating no or small activation of hydrogen excreting microbiota [25], similar in the 0NR mice and the control 30NR mice (Fig. 1D). Methane excretion was at detection levels in both dietary groups (data not shown). Taken together, the diet without vitamin B_3_ did not compromise whole body metabolism nor, more specifically, metabolic flexibility in the female mice.

### Triacylglycerol metabolism

**Fig. 2:**
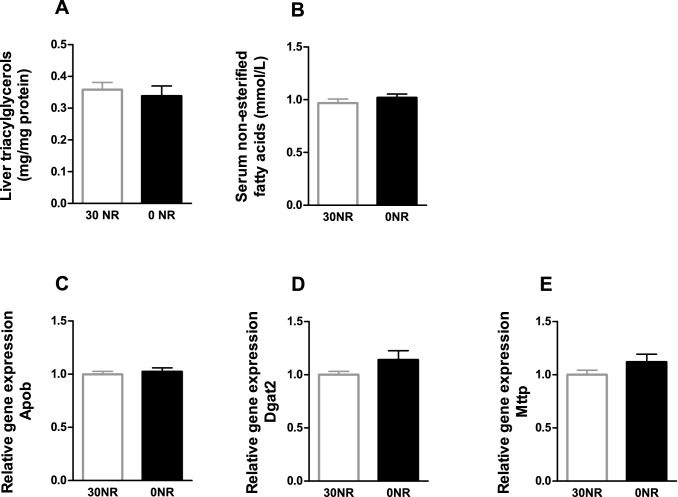
Triacylglycerol metabolism in mice without vitamin B_3_ in the diet (0NR) compared to mice fed 30 mg NR/kg diet (30NR) at the end of the study (*t* = 18 weeks). **a** Triacylglycerols measured in liver, **b** Non-esterified fatty acids measured in serum, and hepatic gene expression: **c** Apolipoprotein B (Apob), **d** Diacylglycerol O-acyltransferase 2 (Dgat2), **e** Microsomal triglycerol transfer protein (Mttp). Values are means ± SEM (*n* = 12)

Supplementation of NR was shown to induce positive effects towards a healthier lipid profile. To analyse the hypothesised negative effects on health induced by depletion of NR, we analysed the triacylglycerol metabolism in the two groups of mice. Serum free fatty acids and liver triacylglycerols were not different in the 0NR mice compared to the 30NR mice, indicating no difference in the uptake and excretion of fatty acids in the liver of the female mice (Fig. 2A and C). Similarly, no differences in gene expression of hepatic *Apob*, *Dgat2*, and *Mttp* were found (Fig. 2D–F).

### White adipose tissue genes responsive to mild vitamin B_3_ deficiency

**Fig. 3:**
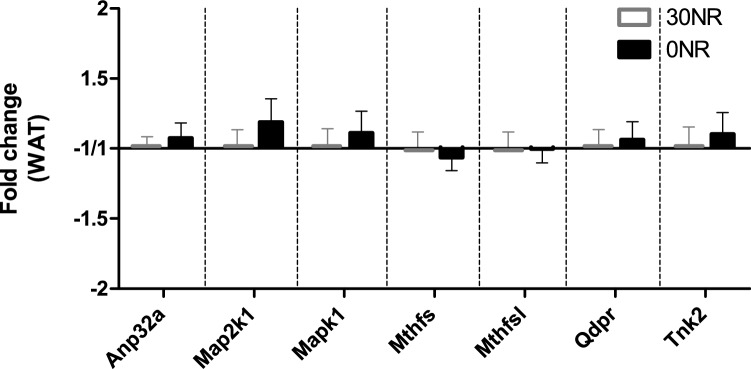
Expression of seven vitamin B_3_ responsive genes in white adipose tissue at the end of the study (*t* = 18 weeks), between control mice with a diet of 30 mg NR/kg diet (30NR) and 0 mg NR/kg diet (0NR). Anp32a = acidic (leucine-rich) nuclear phosphoprotein 32 family, member A, Map2k1 = mitogen-activated protein kinase kinase 1, Mapk1 = mitogen-activated protein kinase 1, Mthfs = 5, 10-methenyltetrahydrofolate synthetase, Mthfsl = 5, 10-methenyltetrahydrofolate synthetase-like, Qdpr = quinoid dihydropteridine reductase, Tnk2 = tyrosine kinase, non-receptor, 2. Values are means ± SEM (*n* = 12)

We previously published the differential expression of seven genes in white adipose tissue of mild vitamin B_3_ deficient male mice [17]. In contrast to what we observed for the male mice, the expression profile of these seven genes in white adipose tissue of the 0NR female mice of the current study was not different from the control 30NR female mice (Fig. 3).

### NAD^+^ and its metabolites

**Fig. 4:**
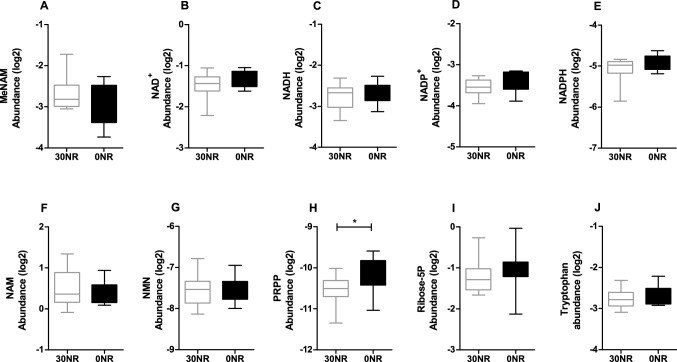
NAD^+^ metabolite abundances measured in liver of control mice on a 30 mg NR/kg diet compared to mice on a 0 mg NR/kg diet (*t* = 18 weeks). **a** Methylnicotinamide (MeNAM), **b** nicotinamide adenine dinucleotide (NAD), **c** NADH, **d** NADP, **e** NADPH, **f** nicotinamide (NAM), **g** nicotinamide mononucleotide (NMN), **h** phosphoribosyl pyrophosphate (PRPP), **i** Ribose-5 phosphate (ribose-5P), **j** Tryptophan. Significant differences of *p* < 0.05 are indicated with *. Values are means ± SEM (n = 12)

To determine whether the absence of NR intake would affect the NAD^+^ metabolite profile, we measured the abundance of several NAD^+^-related metabolites in the liver. No differences between the dietary groups were found in methyl-nicotinamide (MeNAM), NAD^+^ or its reduced form NADH, oxidised or reduced nicotinamide dinucleotide phosphate (NADP^+^ and NADPH), or nicotinamide (NAM) (Fig. 4A–F). Importantly, the levels of NMN, a key metabolite involved in NAD^+^ recycling from NR, which was decreased in livers of male mice on the same diet, was not different between the 0NR and 30NR female mice (Fig. 4G). The phosphoribosyl pyrophosphate (PRPP) level was increased in the 0NR mice compared to the 30NR mice (Fig. 4H), while no differences between these groups were detected for ribose-5-phosphate or tryptophan (Fig. 4I, J). Altogether, these results suggest that the diets used in this study do not have an impact on NAD^+^ metabolism in female mice.

### Insulin sensitivity

**Fig. 5:**
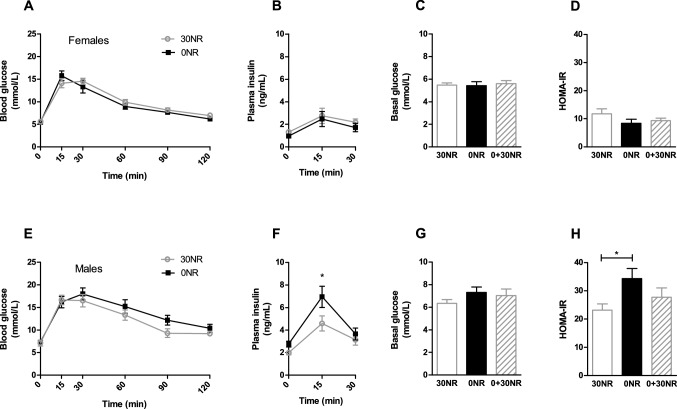
Results of the oral glucose tolerance test and related plasma insulin levels and HOMA-IR of female mice **a**–**d**) fed a diet containing the recommended dose of 30 mg NR/kg diet (30NR), no NR/kg of diet (0NR), or no NR for 15 weeks followed by 3 weeks of 30 mg/kg diet (0 + 30NR); for comparison the data of the male mice **e**–**h**) has been added (published in [17], permission for reproduction granted). **a** and **e** Oral glucose tolerance test, **b** and **f** plasma insulin levels during the oral glucose tolerance test in week 17 at timepoints 0, 15 and 30 min, *n* = 4–7 for females *n* = 11–12 for males, **c** and **g** basal glucose levels, **d** and **h** calculated HOMA-IR, using fasted (basal) glucose and insulin levels (*t* = 0). Values are means ± SEM, *n* = 12 unless stated otherwise)

As female mice did not show differences in white adipose tissue expression of seven genes that were found to be responsive in males upon the same dietary intervention, and since NMN levels were not affected in the female mice, we evaluated insulin sensitivity in the female deficiency model, as this was the most important physiological parameter of male mild vitamin B_3_ deficiency. Fasting blood glucose levels were not significantly different between 30 and 0NR female mice (Fig. 5A/C), neither were circulating glucose levels during the oral glucose tolerance test in female mice on the 0NR diet, compared to female mice the 30NR diet after a glucose bolus (Fig. 5A). Similarly, in the female mice fasting circulating insulin levels and circulating insulin levels during the oral glucose tolerance test were not different between the 30NR and 0NR groups (Fig. 5B). Fasting circulating glucose and insulin levels and circulating glucose response during the oral glucose tolerance test were also not different in male mice on 30NR and 0NR (Fig. 5E–G, [17]). However, the circulating insulin response in the male mice was significantly different in the 0NR compared to the 30NR group (Fig. 5F, [17]). The calculated HOMA-IR, a surrogate marker for insulin sensitivity, was not different between the 0NR and 30NR female mice (Fig. 5D), while previously a clear significant worsening of HOMA-IR was noticed in the 0NR male mice compared to the control 30NR group (Fig. 5H, [17]). Furthermore, while the glucose transporter *Glut4* (*Slc2a4*) tended to be lower expressed in the 0NR male mice compared to the 30NR male mice (FC =  − 1.49, *p* = 0.07, [17]), *Glut4* tended to be slightly higher expressed in white adipose tissue of the 0NR female mice compared to the 30NR female mice (FC = 1.09, *p* = 0.09).

To confirm deficiency status in females and males, we switched an additional group of mice after 15 weeks on the 0NR diet for 3 weeks to the control 30NR diet. In female mice, the 0 + 30NR group did not show any significant differences compared to continuous feeding with 30 mg NR/ kg diet (30NR). In contrast, the worsening of HOMA-IR values of the male mice after 18 weeks on the 0NR diet compared to that of the male mice on the 30NR diet, was prevented in the 0 + 30NR group, which displayed a HOMA-IR comparable to that of the control 30NR group (Fig. 5H). Together, our data indicate that female mice, different from male mice on the same diet, were not vitamin B_3_ deficient in this study.

## Discussion

Female mice that were given a diet without vitamin B_3_ were not different from mice fed the recommended level of vitamin B_3_ regarding body composition, energy metabolism, metabolic flexibility, hepatic triacylglycerols and circulating free fatty acids. Next to this, the expression in white adipose tissue of seven genes, responding to mild vitamin B_3_ deficiency, were not differentially expressed in female mice with NR or without NR in their diets. Hepatic NMN levels, which were lower in livers of male mice on the same vitamin B_3_ deficient diet for the same period, were not affected in the female mice. Lastly, the female mice on the 0NR diet did not have a higher HOMA-IR value compared to the female mice on the 30NR diet, in contrast to the male mice that were fed the 0NR diet for the same duration which showed a higher HOMA-IR value, indicating a higher insulin resistance, compared to male mice on the 30NR diet. The worsening of the HOMA-IR value in the 0NR males was not seen when the mice were given 30NR during the last 3 weeks (0 + 30NR), supporting that the adverse HOMA-IR values in 0NR males were due to vitamin B_3_ deficiency. There were no effects observed in the female mice. Together, these data show that female mice are more resistant to vitamin B_3_ deficiency, compared to male mice of the same strain under the same conditions.

### Tryptophan levels

Investigating effects of (pure) vitamin B_3_ deficiency is complicated by the fact that tryptophan can serve as an alternative source for NAD^+^ [26]. Tryptophan, being an essential amino acid, cannot be left out of a diet as it is not only functional in the de novo synthesis of NAD^+^, but also serves an essential function in protein synthesis and serotonin metabolism [27]. For example, complete depletion of tryptophan from the diet for 32 days led to decreased body weight and decreased serotonin neurotransmission [28]. While the recommended amount of tryptophan in the AIN93 rodent diets is 0.21% for growing animals and 0.16% for adult animals [18], this is an excess that may mask effects of vitamin B_3_ deficiency. The dietary tryptophan level of 0.115% used in this study was chosen to be just above the minimal amount of tryptophan needed for processes unrelated to vitamin B_3_, such as serotonin metabolism, since we wanted to assess vitamin B_3_ deficiency, while maintaining tryptophan sufficiency. The level chosen was based on previous studies; for example, male Fischer-344 rats fed a 0.11% tryptophan diet with no added vitamin B_3_ for 3 weeks seemed healthy, but had decreased NAD^+^ levels in blood, liver and other tissues compared to controls fed a vitamin B_3_ containing diet, showing that this level of tryptophan does affect the NAD^+^ levels without inducing clinical symptoms [29]. Our mice did not show any visible signs of distress, impaired health or, upon dissection, tissue/organ abnormalities, indicating tryptophan sufficiency. This is strengthened by unaffected hydrogen and methane excretion, each of which would be indicative for an altered intestinal microbial activity. An abnormal microbiota activity could indicate a dysbalanced NAD^+^ producing kynurenine pathway, which might be linked to insulin resistance and metabolic syndrome [30]. Also, the glucose and insulin response curves during the oral glucose tolerance test as well as the HOMA-IR values were comparable to that of mice on a similar diet with recommended (AIN93) levels of tryptophan [31]. Together, these results indicate that the 0.115% tryptophan in the diet of the female mice was low, but sufficient. In fact, there is a possibility that the tryptophan levels used in this study are slightly higher than what is absolutely necessary. This could result in a masking effect for vitamin B_3_ deficiency, resulting in no measurable differences between the two groups of female mice, but it would not explain the differences between the female and male mice, which received identical diets.

### NAD^+^ metabolism

Complete or near-complete elimination of NAD^+^ precursors in the diet has severe effects. For example, middle-aged (12-months old) male mice lacking an active nicotinamide phosphoribosyltransferase (NAMPT), enzyme in the salvage pathway, had lower hepatic NAD^+^ levels and showed increased liver triacylglycerol levels, which were restored when the mice were supplemented with NR [32]. Furthermore, *Qprt*^−/−^ mice, deficient in the enzyme quinolinate phosphoribosyltransferase (Qprt) which is involved in the de novo NAD + synthesis pathway, showed lower blood and liver NAD^+^ levels after 23 days, as well as a decrease in body weight within 2 weeks when fed a diet with only tryptophan as a source for NAD^+^, compared to a nicotinic acid containing diet and compared to wildtype mice on either diet as measured in mixed-sex groups [33]. This indicates that depletion of NAD^+^ pools is reached within 2 weeks when neither vitamin B_3_ nor tryptophan is available for synthesis of NAD^+^, while either tryptophan or vitamin B_3_ availability can rescue the deficiency.

Here, in female mice fed a diet with 0.015% tryptophan and without vitamin B_3_ for 18 weeks, hepatic NAD(P)^+^ and NAD(P)H levels were not affected compared to mice fed the same diet containing vitamin B_3_ (Fig. 4). This might be explained by an adaptation effect, since it has been previously reported for rats on a diet with either 0.09% or 0.11% tryptophan that NAD^+^ levels were decreased after 3 weeks, but no longer after 5 weeks [29]. Similar to the females in this study, the male mice in our previous study did not have decreased liver or white adipose tissue NAD^+^ levels after 18 weeks on a diet with 0.115% tryptophan and without vitamin B_3_ [17]. In the male mice a lower level of NMN in the liver was observed in 0NR, compared to 30NR [17], while hepatic NMN levels were indistinguishable between 30 and 0NR in the female mice of this study (Fig. 4). Also, several other NAD^+^-related metabolites measured in the liver were not different between the 0NR and 30NR female mice, except for PRPP, which was increased in the 0NR mice compared to the 30NR control mice. Regretfully, we previously did not analyse PRPP in the male mice. PRPP plays an important role in both the Preiss-Handler pathway and the salvage pathway of NAD^+^ production, as a co-substrate to generate nicotinic acid mononucleotide (NaMN) and NMN, respectively. Therefore, increased PRPP levels could be a compensatory mechanism under circumstances of low NMN or NAD^+^ precursors. Next to its role in NAD^+^ synthesis, PRPP is also required for purine and pyrimidine synthesis, and a relation between purine/pyrimidine synthesis and NAD^+^ production has been established decades ago [34]. Differences in PRPP levels between the 0NR and 30NR female mice of our study could therefore also be related to purine/pyrimidine synthesis, similar to a recent study which showed increased de novo purine synthesis in siRNA transfected NAMPT deficient cells [35]. Overall, our data suggest that the female mouse is more resistant to vitamin B_3_ deficiency, because NR depletion from the diet results in a compensatory PRPP level aligned with an efficient recycling of NAD^+^, keeping NMN levels constant.

### Severity of vitamin B_3_ deficiency is linked to aging, diet and sex

#### Aging

There are several factors influencing the development and the severity of vitamin B_3_ deficiency, one of which is age. Aged female mice were shown to be more susceptible to high-fat diet induced diabetes compared to young females [36], showing that older mice are more vulnerable for e.g. higher blood glucose levels. Next to this, without lowering vitamin B_3_ or tryptophan dietary levels, decreased NAD^+^ levels were found in aged mice of 20–24-months old compared to mice of 4–6 months of age in liver and/ or muscle tissue [32, 37, 38], in 24-months old compared to 3 or 12-months old female Wistar rats in liver, heart kidney and lung [14] and in liver or skin tissue of elderly humans [32, 39]. Furthermore, tryptophan 2,3-dioxygenase levels, involved in the conversion of tryptophan to NAD^+^, was lower in 18-month-old rats, compared to 2–3-months-old rats [40]. Therefore, it may be that the conditions applied in this study were sufficient to maintain vitamin B_3_ status in the relatively young female mice used here, which were 7-months old at the end of the study, while this may not have been the case in old mice with increased needs.

#### Diet

Another NAD^+^ deficiency determining factor is diet and diet-related health. Calorie restriction (30–40%), also increasing longevity, was shown to prevent the age-related decline in muscle NAD^+^ levels of 22-month-old mice [37]. On the other hand, high-fat diet-induced diabetic mice showed impaired glucose tolerance and lower liver and white adipose tissue NAD^+^ levels compared to chow-fed mice [36]. Interestingly, NMN or NR could counteract the age-induced or diet-induced effects on glucose tolerance, NAD^+^ levels, or fat mass [10, 36, 37]. In liver-specific nicotinamide riboside kinase 1 knockout mice, which are unable to metabolise NR in their liver, high-fat diet feeding resulted in declined NAD^+^ levels, which was not seen with low-fat diet feeding, indicating that NR is necessary in challenging situations when there is an increased need for NAD^+^ [41]. As described above, the females in this study did not show any adverse health signs and may thus not be sufficiently challenged to observe effects of the low vitamin B_3_ and tryptophan intakes.

#### Sex

Although most studies in literature have focussed on male mice, this study used female mice. Female mice have been shown to be less prone to the development of diet-induced obesity and insulin resistance; males have different expression profiles of genes involved in the insulin signalling pathway and increased inflammation compared to female mice [19, 20]. Indeed, this study showed lower (basal) glucose and insulin levels during the oral glucose tolerance test, and lower HOMA-IR values in females compared to males fed the same diet (Fig. 5). Next to this, females fed a high-fat diet developed diabetes (based on blood glucose levels) after 6 months, while this was seen in males after 3.5 months [36]. On a normal (chow) diet about 15% of 15–26-months-old male mice developed age-related diabetes, while this was hardly seen in females [36]. Also, glucose tolerance was completely rescued by NMN administration in diabetic female mice, while in male mice this rescue effect was partial. These results are in line with our results, where male mice on the vitamin B_3_ deficient diet showed insulin resistance, a mildly impaired metabolic flexibility and differential expression in white adipose tissue of seven vitamin B_3_ responsive genes [17], while females fed the same period the same 0NR diet did not display these adverse health signs; their insulin sensitivity (Fig. 5), metabolic flexibility (Fig. 1), and white adipose tissue gene expression of the seven vitamin B_3_ responsive genes (Fig. 3) was comparable to these parameters in the 30NR control female mice.

## Conclusion

In conclusion, the female mice in this study did not develop any sign of vitamin B_3_ deficiency after 18 weeks on a 0.115% tryptophan containing diet without vitamin B_3_, being resistant to development of vitamin B_3_ mild deficiency. We propose that the development and severity of a vitamin B_3_ deficiency is linked to age, (dietary) health status and sex. This study particularly shows that, to gain valid research data, we should focus our research on both sexes.

In prospect, as more evidence is becoming available linking vitamin B_3_ deficiency to age-related health problems such as dementia, possibly even linked to an early life vitamin B_3_ deficiency affecting brain development [42], mild, subclinical or temporary vitamin B_3_ deficiency could have more detrimental effects on health than we are aware of. Thus, exploring vitamin B_3_ deficiency, including development of good models and detection methods for males and females, seems to be of great importance.

## Supplementary Information

Below is the link to the electronic supplementary material.

Supplementary file1 (PDF 720 KB)

## Data Availability

All data and materials support their published claims and comply with field standards.
