# Supplementary material for: The female mouse is resistant to mild vitamin B3 deficiency
Source: Eur J Nutr. 2021 Aug 2;61(1):329–40. doi: 10.1007/s00394-021-02651-8 (PMC8783905; doi:10.1007/s00394-021-02651-8)
Supplement: Supplementary file 1 — Supplementary file1 (PDF 720 KB) [file 394_2021_2651_MOESM1_ESM.pdf]

Effects of a vitamin B3 deficient diet on female mice, Inge van der Stelt, Wenbiao Shi, Melissa Bekkenkamp-Grovenstein, Rubén Zapata-Pérez, Riekelt H. Houtkooper, Vincent C.J. de Boer, Maria A. Hegeman, Jaap Keijer (jaap.keijer@wur.nl), European Journal of Nutrition

Supplementary material

Supplementary Table 1. Composition of diets

**Ingredients (g·kg-1 diet)**	**30NR**	**0NR**
Casein	100.0	100.0
Wheat starch	233.1	233.1
Gelatin (hydrolysed)	100.0	100.0
Maltodextrin	100.0	100.0
Sugar	100.0	100.0
Dextrose	50.0	50.0
Arbocel B800	50.0	50.0
Linseed oil	4.0	4.0
Palm oil	206.0	206.0
Mineral mixture AIN-93*	35.0	35.0
Vitamin mixture AIN-93*	10.0	10.0
L-cystine	3.25	3.25
L-phenylalanine	4.32	4.32
Choline chloride 50%	1.55	1.55
Nicotinamide riboside (mg·kg-1 diet)	30.0	0.0
Calculated amount of L-tryptophan (%)	1.15	1.15
Calculated energy (kcal·kg-1)	4653	4653
Carbohydrate (% of total energy)	41	41
Fat (% of total energy)	40	40
Protein (% of total energy)	19	19

* Contains no vitamin B_3 but sufficient minerals and the other vitamins, including iron, vitamin B1, B2 and B6 at the recommended AIN93 levels (Reeves et al., 1993)

Supplementary Table 2. RT-qPCR primer sequences.

**Gene name**	**Forward primer**	**Reverse primer**	**Tissue**
Mttp	GTGGAGGAATCCTGATGGTGA	TGATCTTAGGTGTACTTTTGCCC	Liver
Dgat2	CTATTTGGCTACGTTGGCTGGTAAC	TTGGAGAGCAAGTAGTCTATGGTGTC	Liver
ApoB	TACTTCCACCCACAGTCCCC	AGGGAGCCTAGCAATCTGGA	Liver
Anp32a	CCGTGGGTTCGGGGTTTATT	CTCTTTCACATCAGAGGGCGT	WAT
Map2k1	TGGGCACGAGATCCTACATG	TGGCATCAGGAGGAGGAATG	WAT
Mapk1	ATGACCCAAGTGATGAGCCC	AGCCCTTGTCCTGACCAATTT	WAT
Mthfs	GTCTCTCCTCCTCACGCAGA	GGTCAAGTCCACCAGTGGATAA	WAT
Mthfsl	CGGTGCTTGCCAGGAATG	GCAATCACCTTCTGCGTGAG	WAT
Qdpr	CATTGCTGTGCTCCCCGTTA	TCCTGAGTTTGGCCGTTTGT	WAT
Slc2a4 (glut4)	CCATTCCCTGGTTCATTGTG	GTTTTGCCCCTCAGTCATTC	WAT
Tnk2	ACTTTGGGCTGATGCGAGCACT	AAGGTGCGTGTCTTCAGGCTCT	WAT
Canx	GCAGCGACCTATGATTGACAACC	GCTCCAAACCAATAGCACTGAAAGG	Liver
Hprt 1	TGACACTGGTAAAACAATGCAAACTTTG	GAGGTCCTTTTCACCAGCAAGCT	Liver
Rps 15	CGGAGATGGTGGGTAGCATGG	ACGGGTTTGTAGGTGATGGAGAAC	Liver, WAT
B2m	CCCCACTGAGACTGATACATACGC	AGAAACTGGATTTGTAATTAAGCAGGTTC	WAT

Annealing temperature was 60ºC. Underlined genes are used as reference genes.
